# Correlation of serum PTH level and fracture healing speed in elderly patients with hip fracture

**DOI:** 10.1186/s13018-019-1413-5

**Published:** 2019-11-12

**Authors:** Zhao-Nan Ban, Zheng-Jiang Li, Qi-Shan Gu, Jun Cheng, Qiang Huang, Shu-Xing Xing

**Affiliations:** Department of Orthopedics, Chengdu Fifth People’s Hospital, No.33 Ma Shi Street, Wen jiang District, Chengdu, 611130 Sichuan Province People’s Republic of China

**Keywords:** Elderly patients, Hip fracture, PTH, Fracture healing

## Abstract

**Purpose:**

To access serum parathyroid hormone (PTH) level in elderly patients with hip fracture in relation to fracture healing outcomes.

**Methods:**

This study included 90 elderly male patients with hip fracture and they were defined as the hip fracture group, and they were divided into healing effective group and delayed healing group by final fracture healing outcomes, 45 cases in each group; another 45 male patients older than 70 years without established osteoporosis and hip fracture were included as the control group. The levels of serum PTH level were examined in each group.

**Results:**

Serum PTH level was significantly higher in healing effective group patients at the 7 days and 14 days after fracture than the delayed healing patients.

**Conclusions:**

Our results show that serum PTH level may be an effective indicator of hip fracture delayed healing risk in the elderly.

## Introduction

The main causes of senile osteoporosis include low levels of sex hormones and reduced synthesis of bone metabolism. Osteoporosis and frailty fractures are major public health issues with huge socio-economic costs [1–4]. It has been reported that changes in serum sex hormone levels increase the risk of fracture [5]. Also, lack of vitamin D results in an increase in parathyroid hormone (PTH) levels, leading to bone loss [6, 7]. High bone turnover is associated with an increased risk of bone loss and also associated with an increased risk of fractures [6, 8, 9] and independent of bone mineral density [6, 10–12]. This study examined serum PTH levels in elderly male patients with hip fracture at different times and explored its relationship with fracture healing.

## Material and methods

### Clinical data of patients

This is a retrospective analysis. This group with a total of 90 elderly male patients with hip fracture, aged 70 to 75 (72.6 ± 7.5) years (retired age with better physical condition) was divided into healing effective group and delayed healing group by final fracture healing outcomes, 45 cases in each group. Because of the large number of non-delayed union fracture cases, we randomly selected patients who met the inclusion criteria at the same time from the case series for matching. Another 45 male patients older than 70 years without established osteoporosis (no history of osteoporosis and no anti-osteoporosis treatment) and hip fracture were included as the control group, aged from 70 to 75 (71.8 ± 6.9) years. Naturally, we let an uninformed nurse to extract the patient cases to avoid biases, and she did not know the patient’s examination results and prognosis. Patients with renal diseases, hepatobiliary diseases, other bone diseases, and some drugs that effect to parathyroid hormone level were excluded. All blood samples were obtained in the morning before breakfast. The fracture which not reached the standard of complete healing of fracture within 4 months was defined as bone delayed healing. The study was approved by the Ethics Review Committee of Chengdu Fifth People’s Hospital, and all patients have signed the informed consent.

### Measurement method

The venous plasma of patients in each group were collected at 1 day, 7 days, and 14 days after fracture. Serum levels of PTH were measured and averaged. The normal control group in the calm state by the same period of time for extraction of venous blood also takes the average calculation. Also, urea nitrogen (BUN), creatinine, aspartate transaminase (AST), alkaline phosphatase (ALP), albumin, calcium, phosphate, and total vitamin D were measured in the same sample.

### PTH levels

Using enzyme-linked immunosorbent assay (ELISA), Human PTH ELISA kit were purchased from Aviva Systems Biology (OKEH00642), according to the kit instructions; samples were centrifuged to remove impurities and fibrinolytic block; and 100 uL serum was taken for each sample. The ELISA experiment is operated in accordance with the specification process, and the color intensity is measured at 450 nm.

### Statistical analysis

The data were processed by Statistical Product and Service Solutions (SPSS) 21.0 software, and the serum PTH level was expressed as mean ± SD. One-way analysis of variance (ANOVA) and Student-Newman-Keuls (SNK) were used for comparison. A probability where *p* < 0.05 was considered significant for all statistical comparisons.

## Results

**Fig. 1:**
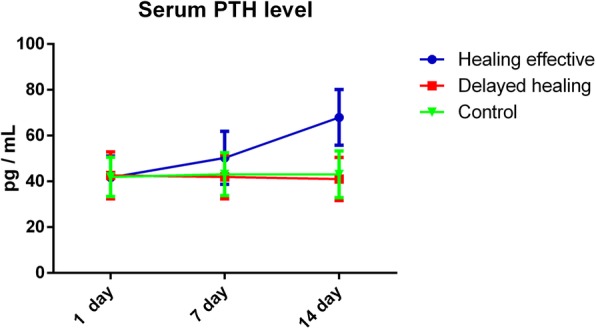
PTH concentration in serum at the 1 day, 7 days, and 14 days after fracture in each group

At the first day after fracture, serum PTH levels in patients with fracture have no significant difference compared with control group (Table 1); at 7 days after fracture, the level of serum PTH in patients from healing effective group was significantly higher than that of delayed healing group (*p* = 0.026) (Fig. 1), but there was no significant difference between each fracture group; at 14 days after fracture, the level of serum PTH in patients from healing effective group was significantly higher than that of delayed healing group (*p* = 0.017) (Fig. 1), and compared with the control group, the difference was significant (*p* = 0.021) (Fig. 1), but there was no significant difference between patients in delayed healing group and control group; serum PTH level in the healing effective group had increased in the above two time points; however, no significant difference was found in delayed healing group and the control group during the above two time points. The changes of serum PTH levels at different times after facture were presented in Table 1.

**Table 1:** The changes of serum PTH levels at different times after facture

Group	1 day	7 days	14 days
Healing effective	41.73 ± 9.34	50.29 ± 11.62^#^	67.92 ± 12.17*^#^
Delayed healing	42.61 ± 10.26	41.93 ± 9.58	41.02 ± 9.42
Control	41.95 ± 8.57	43.15 ± 9.37	43.05 ± 10.20

(*n* = 45, pg/mL, mean ± SD)

Compared with the control group: ^*^p < 0.05

Compared with the control group and delayed healing group: ^#^s*p* < 0.05

## Discussion

Fracture healing process and different from that of other tissues and organs, not only for a long time, involved in many cells and in different healing stages have different cells and cytokines, the healing mechanism is more complicated. Androgen plays an important role in normal bone growth and regulation. Clinical studies have shown that androgen can stimulate the proliferation and differentiation of osteoblasts, increase the cytoplasm of the cells, and increase RNA and protein synthesis, and androgen can promote the secretion of growth hormone. Elderly fracture patients are often prone to Euthyroid Sick Syndrome (ESS); ESS showed that patients had abnormal thyroid hormone levels, but no thyroid disease performance. Elderly fracture patients with ESS present with low thyroid hormone levels and are associated with the severity of the disease. It is also reported that thyroid hormone monitoring is of great significance for the rehabilitation of patients undergoing orthopedic surgery [13, 14]. Parathyroid hormone secreted by the parathyroid gland consists of 84 amino acid residues. Its physiological function is to regulate calcium and phosphorus metabolism in the body. Therefore, high PTH regulates the production of adiponectin in the adipose tissue [15]. Intermittent low-dose recombinant human PTH promotes bone healing by upregulating the expression of mRNA and protein levels of the osteogenic gene Runx2 in the early stages of fracture healing [16]. Parathyroid hormone has been used to treat osteoporosis and reduce the risk of fracture. Animal studies on different fracture models have shown that the addition of PTH promotes fracture healing, increases the volume of the epiphysis and the mineral salt content of the fracture zone, and increases traction resistance [17, 18]. In human studies, PTH has a positive effect on fracture healing, and it seems to affect the role of bone growth factor. A prospective study of 32 patients with nonunion fracture healing from Kastirr et al. found that 95% nonunion patients treated with PTH eventually healed after treatment [19]. We found that PTH levels in healing effective group were higher than those in delayed healing group at the 7 and 14 days after fracture, indicating the role of PTH in fracture healing. PTH is the first bone anabolic drug approved for the treatment of osteoporosis, preventing the delay of fracture healing due to aging [20, 21]. In some high-risk groups, such as the elderly, malnutrition, and postmenopausal women, PTH has better prospects for fracture treatment.

## Conclusion

Serum PTH level was significantly higher in healing effective group patients at the 7 days and 14 days after fracture than the delayed healing patients. Our results show that serum PTH level may be an effective indicator of hip fracture delayed healing risk in the elderly.

## Data Availability

Not applicable

## Abbreviations

ALP: Alkaline phosphatase
ANOVA: One-way analysis of variance
AST: Aspartate transaminase
BUN: Urea nitrogen
ELISA: Enzyme-linked immunosorbent assay
PTH: Parathyroid hormone
SNK: Student-Newman-Keuls
